# Detection of Testosterone Esters in Urine: Evidence of Trace‐Level Detectability in Doping Control Samples

**DOI:** 10.1002/dta.70107

**Published:** 2026-06-17

**Authors:** Daniel Pecher, Guro Forsdahl, Biljana Stojanovic, Günter Gmeiner

**Affiliations:** ^1^ Seibersdorf Labor GmbH Seibersdorf Austria; ^2^ Department of Pharmacy UiT The Arctic University of Norway Tromsø Norway

**Keywords:** doping analysis, Girard P derivatization, LC‐MS/MS, testosterone esters, urine detection

## Abstract

The detection of exogenous testosterone (T) remains a critical challenge in antidoping analysis due to its identical chemical structure to endogenous testosterone. Testosterone is often administered as synthetic esters (T‐esters), which are not naturally produced by the human body and serve as unequivocal evidence of doping. Although T‐esters are traditionally detected in blood samples, their presence in urine has been largely unexplored due to their apolar nature and rapid hydrolysis. In this study, we developed and validated an ultrasensitive LC‐MS/MS method capable of detecting T‐esters in urine at sub–pg/mL concentrations. The method employs derivatization with Girard P reagent to enhance sensitivity and selectivity. Application of the method to doping control urine samples revealed the presence of T‐esters in cases with elevated testosterone/epitestosterone (T/E) ratios, providing what is, to our knowledge, the first evidence of trace‐level detectability of T‐esters in human urine. This novel approach offers a complementary tool for identifying exogenous testosterone use.

## Graphical abstract

An ultrasensitive LC‐MS/MS method for detecting testosterone esters (T‐esters) in urine at sub–pg/mL levels is presented. This approach, employing Girard P derivatization, provides the first evidence of T‐esters' trace‐level detectability in human urine. The method offers a complementary tool for identifying exogenous testosterone use, advancing antidoping science.

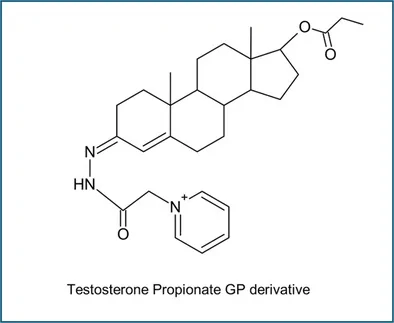

## 1 Introduction

The use of exogenous testosterone (T) remains one of the most commonly abused forms of doping [1]. Reliable discrimination between endogenous and exogenous testosterone is, however, complex due to their identical chemical structure. Today, the detection of testosterone intake can be achieved through different approaches in doping analysis. One approach is the use of the steroid passport [2], which monitors an athlete's steroid profile in urine over time. This method allows for the detection of abnormal variations in endogenous steroid levels, which may indicate the use of anabolic steroids. Urine samples showing irregularities can then be further analyzed using a combination of gas chromatography and isotope ratio mass spectrometry (GC‐IRMS) [3]. This approach has limits, however, because preparations available on the black market do not necessarily differ significantly in their carbon isotope ratio from endogenously produced T [4].

Testosterone is commonly administered intramuscularly as prodrug formulations in the form of esters with various chain lengths [5, 6]. In the context of doping in sports, alternative formulations such as transdermal testosterone preparations or oral testosterone undecanoate are also likely to be misused due to their comparatively short detection windows. Testosterone esters (T‐esters) are synthetic compounds that are not naturally produced by the human body, and their presence in an athlete's sample is considered clear evidence of doping and automatically leads to an Adverse Analytical Finding (AAF). This means that the detection of T‐esters unequivocally indicates the use of prohibited substances, as they cannot be attributed to endogenous production.

The detection of testosterone esters is currently performed primarily in blood samples, such as serum or plasma, or in dried blood spots [7, 8, 9, 10, 11, 12, 13, 14, 15, 16]. It has generally been assumed that T‐esters are not excreted in urine in detectable amounts due to their apolar nature and the activity of esterases [17]. To the best of our knowledge, no studies to date have investigated the presence of T‐esters in urine at trace levels.

Therefore, the aim of the present study was to develop an ultrasensitive HPLC‐MS/MS method capable of detecting T‐esters in urine samples at low to sub–pg/mL concentrations. Additionally, the method was applied to analyze samples from individuals suspected of T‐ester administration. For increased sensitivity of the analysis, the method is based on the derivatization of the T‐esters with Girard P agent [17].

## 2 Material and Methods

### 2.1 Chemicals and Reagents

Testosterone (T), testosterone acetate (T‐acetate), testosterone benzoate (T‐benzoate), testosterone cypionate (T‐cypionate), testosterone decanoate (T‐decanoate), testosterone enanthate (T‐enanthate), testosterone isocaproate (T‐isocaproate), testosterone laurate (T‐laurate), testosterone phenylpropionate (T‐phenylpropionate), testosterone propionate (T‐propionate), testosterone undecanoate (T‐undecanoate), testosterone valerate (T‐valerate), and testosterone hexahydrobenzoate (T‐hexahydrobenzoate) were obtained from Steraloids (Newport, RI, United States). Methanol (HPLC grade), used for preparing standard solutions, and n‐pentane (HPLC grade) were purchased from Chem‐Lab (Zedelgem, Belgium).

Deuterium labeled (d6) T‐acetate was synthesized in‐house by dissolving 1 mg of d3‐testosterone in 250 μL of dry pyridine and 250 μL of d6‐acetic anhydride. The reaction mixture was heated for 10 min at 60°C, followed by evaporation to dryness. The residue was reconstituted in 10 mL of methanol to obtain a stock solution.

Milli‐Q water, obtained from a water purification system (Millipore, Burlington, MA, United States), was used for buffer preparation. Potassium dihydrogen phosphate, di‐sodium hydrogen phosphate, and n‐pentane were sourced from Merck (Darmstadt, Germany). Methyl‐t‐butyl‐ether (MTBE) and trifluoroacetic acid (TFA) were obtained from Sigma Aldrich (St. Louis, MO, United States), and GP reagent (1‐(carboxymethyl)pyridinium chloride hydrazide) was purchased from Santa Cruz Biotechnology (Dallas, TX, United States). All solvents used for HPLC analysis (ULC/MS‐CC/SFC grade) were supplied by Biosolve (Valkenswaard, the Netherlands).

### 2.2 Sample Preparation

Five milliliters of urine sample was mixed with 10 μL of an internal standard mixture (T‐acetate d6 40 ng/mL), and 1 mL of phosphate buffer (pH 6.8) was added. The analytes were extracted with 5‐mL MTBE. The organic phase was evaporated to dryness, and the samples were reconstituted by the addition of 100 μL of Girard P solution (4 mg/mL, in MeOH:TFA, 95:5 V/V). The analytes were derivatized by placing the extracts on a heating block (60°C) for 10 min. The obtained solution was injected into the online SPE‐LC‐MS system.

### 2.3 Liquid Chromatography–Mass Spectrometry

The analysis was performed on a Vanquish Horizon UHPLC+ System coupled to a TSQ Altis mass spectrometer (Thermo Fisher Scientific, San Jose, CA, United States).

The chromatographic system was equipped with a Kinetex Phenyl‐Hexyl precolumn (10 × 2.0 mm i.d., 2.6‐μm particle size; Phenomenex) and the analytical column InfinityLab Poroshell 120 EC‐C18 (100 × 2.1 mm i.d., 1.9‐μm particle size) (Agilent Technologies, Santa Clara, CA, United States). The mobile phase consisted of water with 0.2% formic acid (Solvent A) and methanol with 0.1% formic acid (Solvent B). A gradient program was employed, starting with 30% B for 2 min, increasing to 65% B over 0.5 min, then ramping to 100% B over 12.5 min. This was followed by an isocratic step at 100% B for 2.5 min, a return to 30% B within 0.1 min, and re‐equilibration at the starting conditions for 2.4 min. The total runtime was 20 min.

The mass spectrometer was equipped with a heated electrospray ionization (ESI) source and operated in positive ionization mode, with the spray voltage set to 3500 V. The ion transfer tube temperature was maintained at 325°C, whereas the vaporizer temperature was set to 350°C. The sheath and auxiliary gas (nitrogen) flow rate was 35 and 10 arbitrary units, respectively. The system was operated in selected ion monitoring (SRM) mode, using argon as the collision gas at a pressure of 1.5 mTorr. Multiple ion transitions were monitored, covering all analyzed esters in SRM mode.

### 2.4 Optimization of the Extraction Procedure

The extraction procedure was optimized by comparing the recoveries obtained by extracting spiked urine samples (1 ng/mL) under varying pH conditions (no pH adjustment, 4.8, 6.8, and 9.8), and using different extraction solvents (MTBE, ethyl acetate, n‐Pentane). The recoveries were calculated as the ratio of peak area obtained for extracted spiked urine samples to peak area obtained for the standard solution prepared at the same concentration level.

### 2.5 Method Validation

The method was validated in accordance with WADA guidelines for nonthreshold substances [18]. The validation parameters included limit of detection (LOD), selectivity, extraction recovery, extract stability, and carryover.

The limits of detection were determined by spiking blank urine samples with standard solutions of the target analytes. Urine samples were prepared at the following concentrations: 0.05, 0.1, 0.25, 1, 2.5, 10, 25, 100, 250, and 1000 pg/mL. Additionally, 10 blank urine samples were prepared to evaluate the method's selectivity and to check for potential interferences. All samples were prepared and analyzed across two separate batches.

Carryover was evaluated by analyzing a spiked sample at a concentration of 200 pg/mL, followed by the analysis of three extracted blank samples.

Extract stability was assessed by reanalyzing six spiked urine samples (50 pg/mL) 72 h after the initial analysis.

### 2.6 Applicability of the Method

The developed method was applied to 13 doping urine samples (Samples 1–13) with high testosterone/epitestosterone (T/E) ratios (ranging between 5.4 and 113), where T abuse was previously confirmed by GC‐IRMS. For one of the samples (Sample 1), an additional aliquot was prepared and reanalyzed using an LC‐MS/MS method further optimized for detection of T‐propionate. All urine samples were obtained from athletes during routine doping control procedures. The samples were anonymized. All athletes provided written consent, implemented in the collection form, for the use of their samples for research and method development purposes. The samples were used in line with the World Anti‐Doping Code [18].

## 3 Results and Discussion

### 3.1 Optimization of the Extraction Procedure

The extraction experiments performed across varying pH conditions and using different organic solvents resulted in recoveries ranging from 47% to 107%. Based on these comparative evaluations, extraction with MTBE at pH 6.8 was identified as the optimal procedure, yielding consistently high recoveries between 85% and 107%.

### 3.2 Method Performance

The method validation demonstrated compliance with WADA guidelines for nonthreshold substances. The limits of detection (LODs) were successfully established, and LOD values for all target analytes are presented in Table 1, together with the selected MS conditions and retention times.

**TABLE 1:** Overview of the selected MS conditions, retention times (RT), and limits of detection (LOD) of 12 testosterone esters included in the developed method.

Substance	Precursor ion (m/z)	Product ions (m/z)	Collision energy (V)	LOD (pg/mL)	Retention time (min)
T‐acetate d6 GP	470	163, 363, 391	48, 36, 32	—	6.03
T‐acetate GP	464	163, 357, 385	39, 29, 26	0.25	6.03
T‐benzoate GP	526	163, 419, 447	40, 30, 28	1.00	8.51
T‐cypionate GP	546	151, 163, 467	42, 43, 30	0.25	10.38
T‐decanoate GP	576	163, 469, 497	43, 32, 30	0.10	11.93
T‐enanthate GP	534	151, 163, 455	37, 39, 26	1.00	10.18
T‐isocaproate GP	520	163, 413, 441	41, 30, 28	1.00	9.29
T‐laurate GP	604	135, 497, 525	47, 33, 31	0.05	12.84
T‐phenylpropionate GP	554	151, 447, 475	40, 31, 28	0.25	8.83
T‐propionate GP	478	151, 163, 399	37, 39, 26	0.25	6.97
T‐undecanoate GP	590	163, 483, 511	43, 33, 30	0.05	12.41
T‐valerate GP	506	151, 163, 399, 427	43, 43, 28, 29	0.10	8.63
T‐hexahydrobenzoate GP	532	151, 163	43, 49	0.25	9.75

**FIGURE 1:**
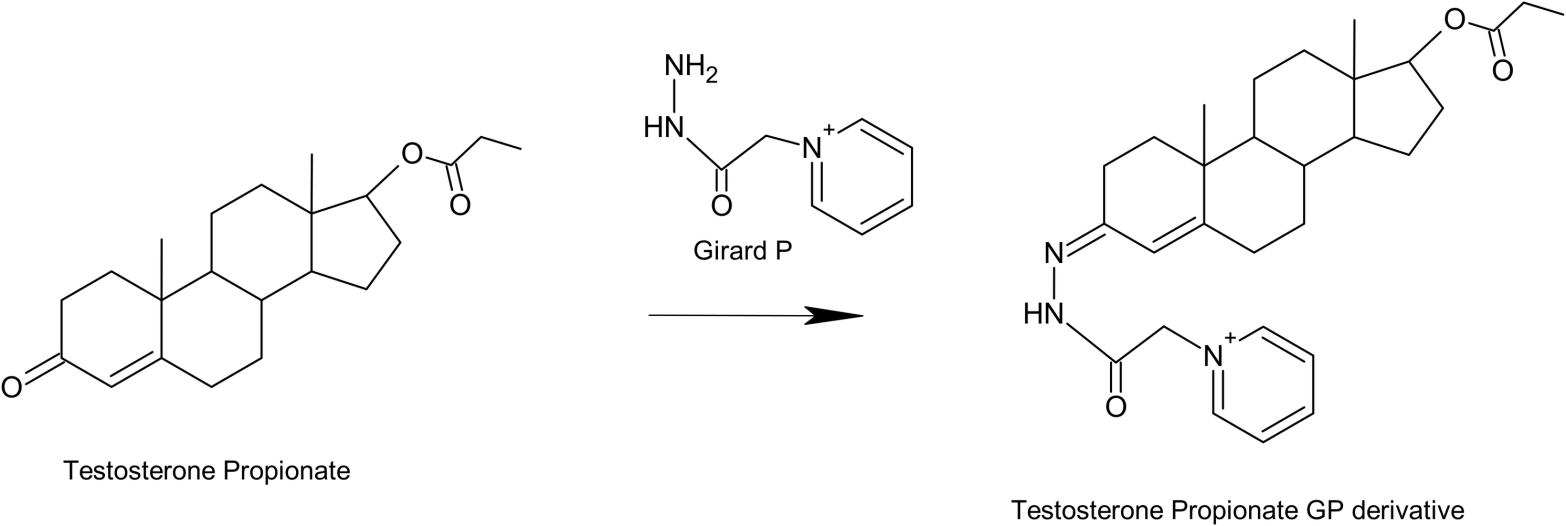
Derivatization of testosterone propionate with Girard P reagent.

The method is based on the derivatization of the T‐esters with the Girard P reagent, as illustrated in Figure 1. The reaction with Girard P allows the formation of hydrazone derivatives. This chemical modification improves the sensitivity of the analysis by increasing the ionization efficiency of the T‐esters, thereby enabling their detection at sub–pg/mL concentrations. Additionally, the use of Girard P derivatization enhances the selectivity of the method by producing unique diagnostic ions, which aid in the accurate identification of T‐esters in complex biological matrices such as urine.

**FIGURE 2:**
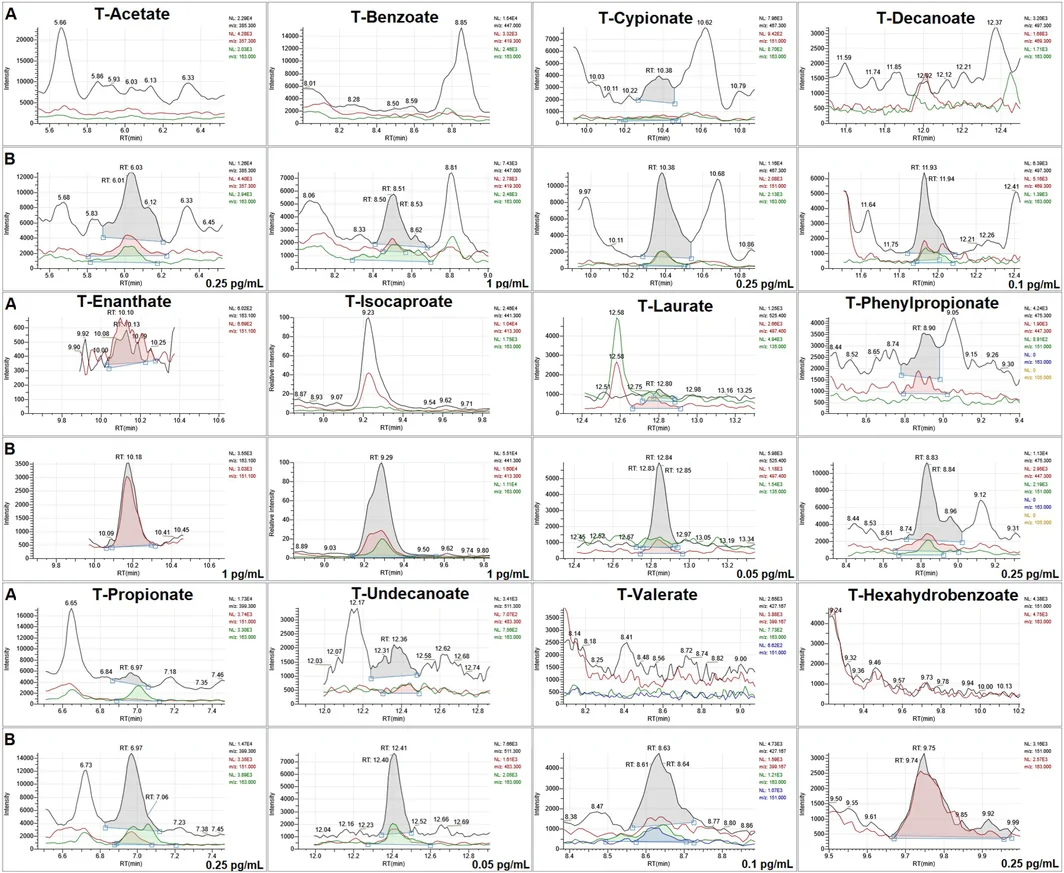
Chromatograms obtained by analysis of blank samples (A rows) and samples spiked at LOD (B rows).

The presented method allows for the simultaneous detection of the presence of 12 T‐esters in urine samples with LODs in the range 0.05–1 pg/mL. Figure 2 presents extracted chromatograms from the analysis of blank urine samples and urine samples spiked at the LOD.

For 11 out of the 12 T‐esters, no significant interferences were observed in the blank urine samples, highlighting the good selectivity of the method. For T‐isocaproate, however, endogenous interferences were detected in two of the three monitored ion transitions at retention times close to that of the analyte. These interfering signals are attributed to an unknown endogenous compound that elutes just before T‐isocaproate under the applied chromatographic conditions. Notably, the third ion transition showed no detectable interference in the blank samples. As a result, the identification of T‐isocaproate remains feasible during the testing procedure, and the overall identification of the analyte is not compromised.

Regarding the detection of exogenous T‐acetate, acetylation is a well‐known metabolic transformation in human physiology. However, it is not a recognized metabolic pathway for steroids, including testosterone [19]. To the best of our knowledge, no evidence exists to suggest that T‐acetate is produced endogenously in humans, even at trace levels. Therefore, the presence of T‐acetate in urine is highly unlikely to result from endogenous processes, implying an exogenous origin. This is further supported by the validation data, which showed no detectable signal for T‐acetate in blank urine samples.

No carryover was detected following the analysis of highly concentrated samples, and the extract stability was confirmed, with analyte concentrations remaining within acceptable limits after 72 h of storage.

Overall, the method was deemed reliable and fit for purpose for the analysis of nonthreshold substances in accordance with WADA requirements.

### 3.3 Reanalysis of Urine Samples Previously Confirmed Positive for Testosterone

**FIGURE 3:**
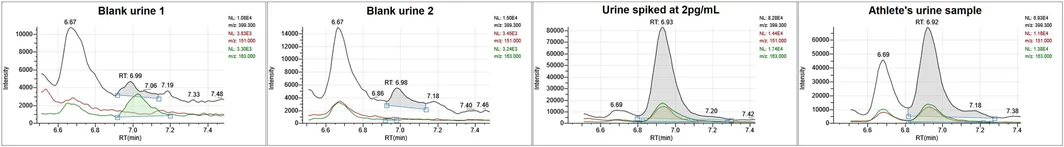
Extracted chromatograms obtained using the developed LC‐MS/MS method, showing the analysis of two blank urine samples, a urine sample spiked with T‐propionate at 2 pg/mL, and a urine sample from an athlete (Sample 1, T/E = 10) with previously confirmed T abuse by GC‐IRMS.

The developed method was applied to 13 doping control urine samples exhibiting elevated T/E ratios, ranging from 5.4 to 113. In these samples, testosterone abuse had previously been confirmed by GC‐IRMS. With the developed method, a Presumptive Adverse Analytical Finding (PAAF) for one or more T‐esters was identified in five of the samples, all of which corresponded to cases with T/E ratios above 10 (T/E 10–113). The detected T‐esters included T‐propionate and T‐enanthate, showing the presence of exogenous T administration. A detailed overview of the T/E ratios and the results of the T‐ester analysis is provided in Table 2. Additionally, the extracted chromatograms for T‐propionate in one of the PAAF cases (Sample 1) are presented in Figure 3.

**TABLE 2:** T/E ratios and results of the T‐ester analysis for 13 athlete samples previously confirmed positive for testosterone abuse by GC‐IRMS.

Samples	T/E ratio	Finding
1	10	T‐propionate
2	19	T‐propionate
3	5.4	—
4	23	T‐propionate
5	11	—
6	14	—
7	113	T‐enanthate
8	15	—
9	21	—
10	42	T‐propionate T‐enanthate
11	20	—
12	17	—
13	7.1	—

To further support this evidence, an additional aliquot of Sample 1, containing a PAAF for T‐propionate, was equally prepared according to the developed procedure and reanalyzed with an optimized gradient (25% B for the first 2 min, followed by linear increase to 100% over 18 min, isocratic step at 100% B for 2.5 min, and re‐equilibration at 25% B for 2.5 min) and addition of two new mass transitions for T‐propionate (478 → 371, ce 29 and 478 → 135, ce 43). In this analysis, the signals comply with the criteria of identification according to the WADA Technical Document TD2023IDCR [19], comparing the retention time (RT) and the relative abundances (RAs) of the analyte's diagnostic ions in the athlete sample with those obtained from a reference sample analyzed within the same analytical batch.

**FIGURE 4:**
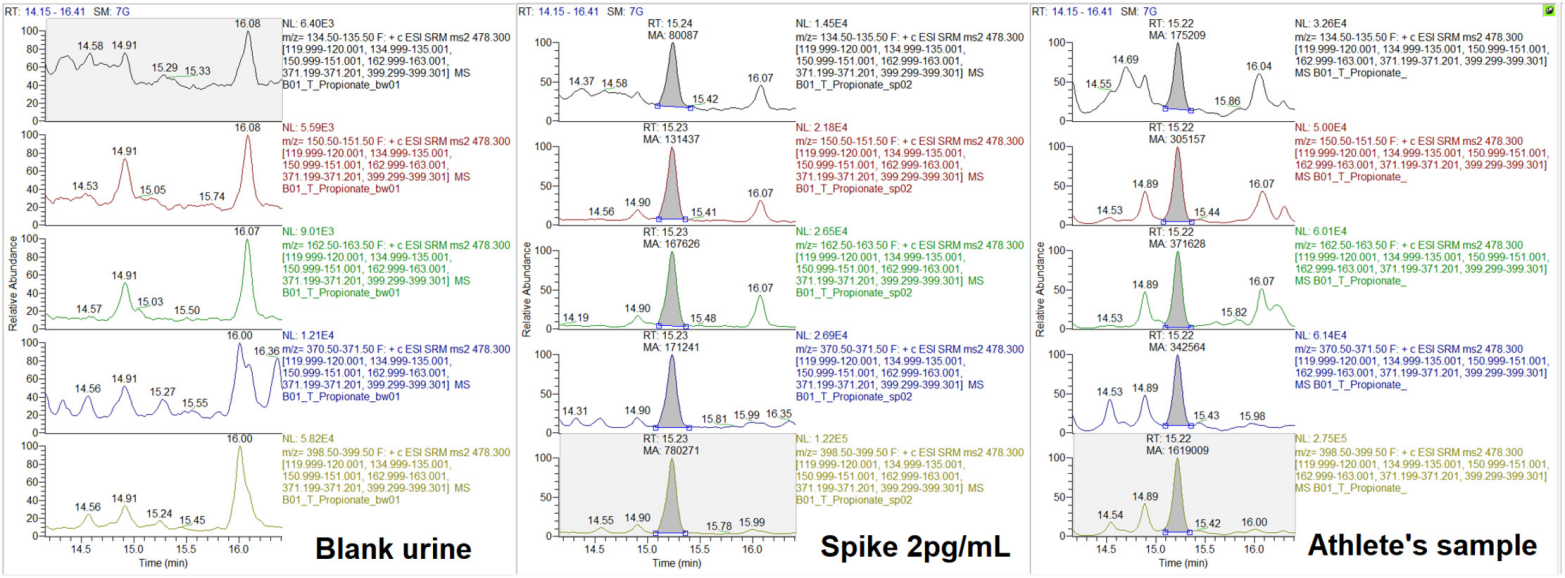
Extracted chromatograms obtained using the LC‐MS/MS procedure for reanalysis, showing the analysis of a blank urine, a urine sample spiked at 2 pg/mL, and a urine sample from an athlete (Sample 1, T/E = 10) with previously confirmed T abuse by GC‐IRMS.

The estimated concentration of T‐propionate was determined to be in the low pg/mL range, demonstrating the sensitivity of the applied method. The results obtained from the LC‐MS/MS confirmation procedure are illustrated in Figure 4, providing clear evidence of the analyte's presence and supporting the applicability of the analytical approach.

## 4 Conclusion

An ultrasensitive LC‐MS/MS method was successfully developed for the detection of T‐esters in urine samples at sub–pg/mL concentrations. The method demonstrated excellent sensitivity, allowing for the detection of T‐esters in multiple doping control urine samples. Importantly, this study provides, to the best of our knowledge, the first evidence of T‐esters being detected in trace amounts in human urine. This represents a major advancement in the field of doping analysis, as it demonstrates that urine samples alone can be used to directly detect T‐esters, particularly in cases where blood samples are unavailable.

To further enhance the specificity of the method and reduce potential interferences, the implementation of an alternative sensitive derivatization approach is recommended. This will be investigated in future studies.

Overall, this study introduces a novel and important analytical approach, contributing to the advancement of T‐ester analysis and antidoping science.

## Author Contributions

**Daniel Pecher:** investigation, methodology, resources, validation, visualization, writing – review and editing. **Guro Forsdahl:** investigation, supervision, visualization, writing – original draft, writing – review and editing. **Biljana Stojanovic:** investigation, methodology, resources, validation, visualization, supervision, writing – review and editing. **Günter Gmeiner:** conceptualization, funding acquisition, project administration, investigation, methodology, supervision, writing – review and editing.

## Funding

This work was supported by the Federal Ministry of Housing, Arts, Culture, Media and Sport of the Republic of Austria.

## Conflicts of Interest

The authors declare no conflicts of interest.

## Data Availability

The data that support the findings of this study are available from the corresponding author upon reasonable request.
